# Methicillin-resistant *Staphylococcus aureus* (MRSA) and anti-MRSA activities of extracts of some medicinal plants: A brief review

**DOI:** 10.3934/microbiol.2019.2.117

**Published:** 2019-04-15

**Authors:** Maureen U. Okwu, Mitsan Olley, Augustine O. Akpoka, Osazee E. Izevbuwa

**Affiliations:** 1 Department of Biological Sciences, College of Natural and Applied Sciences, Igbinedion University Okada, Edo State, Nigeria; 2 Department of Pathology, Igbinedion University Teaching Hospital, Okada, Edo State, Nigeria

**Keywords:** Methicillin-resistant *Staphylococcus aureus* (MRSA), Vancomycin-intermediate *S. aureus* (VISA), Vancomycin-resistant *S. aureus* (VRSA), Staphylococcal Cassette Chromosome mec (SCCmec), anti-MRSA plants

## Abstract

The increasing emergence of multidrug-resistant infection causing microorganisms has become a significant burden globally. Despite the efforts of pharmaceuticals in producing relatively new antimicrobial drugs, they have resulted in a high rate of mortality, disability and diseases across the world especially in developing countries. Supporting this claim was the report of the Centre for Disease Control and Prevention (CDC) who estimated that over 2 million illnesses and 23,000 deaths per year are attributable to antibiotic resistant pathogens in the United States. They include Methicillin-resistant *Staphylococcus aureus* (MRSA), Vancomycin-intermediate *Staphylococcus aureus* (VISA), Vancomycin-resistant *Staphylococcus aureus* (VRSA), Vancomycin-resistant enterococci (VRE), Extended spectrum beta-lactamases (ESBLs) producing gram-negative bacilli, Multidrug-resistant *Streptococcus pneumoniae* (MDRSP), Carbapenem-resistant Enterobacteriaceae (CRE) and Multidrug-resistant *Acinetobacter baumannii*. For MRSA, resistance is as a result of Methicillin-sensitive *S. aureus* (MSSA) strains that have acquired Staphylococcal Cassette Chromosome mec (SCCmec) which carries mecA gene. The gene encodes the penicillin-binding protein (PBP2a) which confers resistance to all β-lactam antibiotics. Vancomycin was previously the widely preferred drug for the treatment of MRSA infections. It is no longer the case with the emergence of *S. aureus* strains with reduced vancomycin sensitivity limiting the conventional treatment options for MRSA infections to very scanty expensive drugs. Presently, many researchers have reported the antibacterial activity of many plant extracts on MRSA. Hence, these medicinal plants might be promising candidates for treatment of MRSA infections. This work is a brief review on Methicillin-resistant *Staphylococcus aureus* (MRSA) and the anti-MRSA activities of extracts of selected medicinal plants.

## 1. Introduction

Multidrug-resistant bacteria (MDRB) are microorganisms that are resistant to one or more antimicrobial agents. They are usually resistant to all but one or two commercially available antimicrobial agents. This definition includes microbes that have acquired resistance to at least one agent in three or more antimicrobial categories. The MDRB of clinical interest include: Methicillin-resistant *Staphylococcus aureus* (MRSA), *Staphylococcus aureus* with resistance to vancomycin [these are Vancomycin-intermediate *Staphylococcus aureus* (VISA) and Vancomycin-resistant *Staphylococcus aureus* (VRSA)],Vancomycin-resistant enterococci (VRE), Extended spectrum beta-lactamases (ESBLs) producing gram-negative bacilli, Multidrug-resistant *Streptococcus pneumoniae* (MDRSP), Carbapenem-resistant Enterobacteriaceae (CRE) and Multidrug-resistant *Acinetobacter baumannii* [1]–[3] .

Infectious diseases caused by MDRB are an important burden globally. They have for centuries been among the leading causes of death, disability, growing challenges to health security and human progress, especially in developing countries [4].

Although, many new antibacterial drugs have been produced, bacteria exhibiting resistance to them have increased and is becoming a global concern as we are fast running out of therapeutic options [5],[6]. The challenges of antimicrobial resistance are faced in both the health care and community settings, necessitating a broad approach with multiple partners across the continuum of care. For example, 18–33% of MRSA colonized patients subsequently developed MRSA infections. Community-acquired methicillin-resistant *Staphylococcus aureus* (CA-MRSA) strains also constitute an increasing proportion of hospital-onset MRSA infections. The Centre for Disease Control and Prevention (CDC) estimated that over 2 million illnesses and 23,000 deaths per year are attributable to antibiotic resistance in the United States [3].

Vancomycin is widely prescribed for the treatment of infections caused by MRSA; but the emergence of VISA and VRSA has been reported by many authors. Really, teicoplanin, daptomycin, linezolid, etc are expensive drugs which are currently prescribed when faced with MRSA with low sensitivity to vancomycin. However, development of resistance to these drugs has been identified worldwide [7]–[11].

Usage of plants in fighting against illnesses and diseases has deep roots in man's history. Researchers are interested in plant extracts as medicines because there are several reports regarding the antimicrobial activity of their crude extracts which might be better substitutes for conventional antibiotics. Recent published reports opined that medicinal plants with anti-MRSA activity can be considered for treatment of MRSA infections [8],[12]. This present work is a brief review on MRSA, VISA, VRSA and some medicinal plants with anti-MRSA activities.

## 2. Emergence and resistance mechanisms of MRSA, VISA and VRSA

### 2.1. MRSA

*Staphylococcus aureus* is a Gram-positive coccoid bacterium. The cells are arranged in irregular grape-like appearance and they are usually found as normal flora in humans and animals. It is ubiquitous in the human population and 30–40% of adults are asymptomatic carriers. It is also a major pathogen of human and can cause a range of infections from mild skin infections and food poisoning, to life threatening infections [13]–[17].

Resistance to methicillin by *S. aureus* was initially observed in 1961 shortly after the antibacterial agent was introduced clinically and since then, there has been a global epidemic of Methicillin-resistant *Staphylococcus aureus* (MRSA) in both healthcare and community settings [18]–[20]. MRSA isolates from the UK and Denmark in the early 1960s constituted the very first epidemic MRSA clone soon after methicillin was introduced and it has since emerged as an important pathogen in human medicine [21]–[23]. Although, methicillin is no longer prescribed for patients and has been replaced by isoxazolyl penicillins, particularly flucloxacillin in the UK, the acronym MRSA has stayed [24]. It is characterized by antibiotic resistance to penicillins, cephalosporins, carbapenems and has tendency of developing resistance to quinolones, aminoglycosides, and macrolides [10],[25],[26].

The origination of MRSA was as a result of Staphylococcal Cassette Chromosome mec (SCCmec) genes acquired by methicillin-susceptible S. aureus (MSSA). The SCC*mec* harbours the mecA gene which encodes the penicillin-binding protein (PBP2a) that confers resistance to all β-lactam antibiotics [10],[27]–[29]. SCC*mec* also contains the cassette chromosome recombinases (*ccr*) gene complex. The *ccr* genes (composed of *ccrC* or a pair of *ccrA* and *ccrB*) encode recombinases mediating integration and excision of SCC*mec* into or from the chromosome. The *ccr* genes and surrounding genes form the *ccr* gene complex. In addition to *ccr* and *mec* gene complexes, SCC*mec* contains a few other genes and various other mobile genetic elements such as: insertion sequences, transposons and plasmids [30],[31].

Eleven different types of SCC*mec* (I-XI) and five allotypes of the *ccr* gene complexes (*ccrAB1*, *ccrAB2*, *ccrAB3*, *ccrAB4* and *ccrC*) have been reported. Generally, SCC*mec* types I, II, III, VI and VIII are called hospital-acquired MRSA or (HA-MRSA). Types IV, V and VII as community-acquired (CA-MRSA) while types IX, X and XI as livestock-associated MRSA (LA-MRSA) [31],[32]. Expression of methicillin resistance in *S. aureus* is commonly under regulatory control by *mec*I or *bla*I gene. The *mec*I and *bla*I repressors are controlled by the *mec*RI and *bla*RI transducers [20].

MRSA remains a major public health concern worldwide and a therapeutic challenge as the antibacterial drugs effective for treatment are scanty and costly. The changing epidemiology of MRSA infections, varying resistance to commonly used antibiotics and involvement in hospital and community infections are influencing the use and clinical outcomes of currently available anti-infective agents [33].

### 2.2. Resistance of vancomycin by S. aureus

Vancomycin is an antibacterial agent that inhibits cell wall production by binding with the D-alanyl-D-alanine C terminus of the bacterial cell wall precursors, and subsequently preventing cross-linking by transpeptidation. Vancomycin acts extracellularly and inhibits late-stage peptidoglycan biosynthesis which results in the intracellular accumulation of UDP-linked MurNAc-pentapeptide precursors. The vancomycin complex involves a number of hydrogen bonds between the peptide component of vancomycin and the D-Ala-D-Ala residue. Any process that interferes with vancomycin binding to D-Ala-D-Ala residues in the cell wall will decrease the potency of the drug [13],[36].

Vancomycin was widely utilized for the treatment of MRSA infections and has led to the emergence of vancomycin-intermediate and vancomycin-resistant *S. aureus* (VISA and VRSA) [37]. This also triggered off alarms in the medical community as *S. aureus* causes life-threatening infections in hospitalized and non-hospitalized patients [38]. Vancomycin-intermediate *S. aureus* (VISA), heterogeneous vancomycin-intermediate *S. aureus* (hVISA) and vancomycin-resistant *S. aureus* (VRSA) are the three classes of *S. aureus* that are resistant to vancomycin which have emerged in different locations of the world [39].

### 2.3. Vancomycin-intermediate S. aureus (VISA)

Vancomycin-intermediate *S. aureus* (VISA) was first reported from Japan in 1996 with reduced susceptibility to vancomycin (having a Minimum Inhibitory Concentration (MIC) of 8 mg/L). It has now spread to other hospitals in Asia, France, Brazil, USA, United Kingdom, etc [40]. *S. aureus* vancomycin breakpoints were redefined by the Clinical and Laboratory Standards Institute (CLSI) in 2006 as follows: resistant at MIC ≥ 16 µg/ml, intermediate at 4–8 µg/ml and susceptible at ≤ 2 µg/ml [34]–[36].

VISA isolates emerged as a result of mutations (not their acquisition of foreign genetic elements) in MRSA isolates during treatment of patients with vancomycin. The comparison of vancomycin-susceptible and -resistant isolates to the VISA isolates showed that the mutations often occurred in the *walkR, vraSR*, *rpoB* (ribosomal) genes and the *yvqF/vraSR* system. Usually, the relevant mutated genes seemed to be directly or indirectly involved with the biosynthesis/metabolism of the staphylococcal cell wall [41].

Often, there were treatment failures when VISA infections were treated with vancomycin [41]. It was observed that under vancomycin selective pressure usually during treatment, the VISA strains with a vancomycin MIC of 8 µg/ml have emerged and led to therapy failure. However, the nature of this resistance phenotype (VISA) was unstable especially when vancomycin selective pressure is removed as some strains reverted back to vancomycin-susceptible strains with MIC at 2 µg/ml [36].

### 2.4. Heterogeneous VISA (hVISA)

In 1997, the first case of hVISA was reported in Japan. The cultures of hVISA strains contain both low-frequency subpopulations of bacteria with increased vancomycin MIC value and high frequency of bacteria with low vancomycin MIC values (close to those of susceptible strains) [41]. The MIC for hVISA strains was defined by the presence of subpopulations of VISA at a rate of one organism per 105 to 106 organisms [42],[43]. The hVISA strains were detected using vancomycin population analysis profile (PAP) which was proposed as the most accurate method for hVISA detection; however, it is relatively time-consuming and requires the use of a spiral plater. The hVISA strain has generally required formal population analysis using the serial passage of screened isolates of *S. aureus* on selective agar containing increasing concentrations of vancomycin for its detection [13]. Results are generally not ready until at least 3 to 5 days [36].

VISA and hVISA strains have thickened cell wall with reduced glycopeptide cross-linking as a result of the complex reorganization of cell wall metabolism. It has been proposed that the thickened cell wall may trap and sequester vancomycin and consequently, interferes with its mode of action [13]. This could be due to alteration in peptidoglycan production leading to increased residues of D alanyl-D-alanine, which bind vancomycin molecules and prevent them from reaching the target sites [18]–[20].

### 2.5. Vancomycin-resistant S. aureus (VRSA)

In 2002, the first hospital strain of Vancomycin-resistant *S. aureus* (VRSA) was reported in the United States [44]. The acquisition of *vanA* gene from vancomycin-resistant enterococci resulted in the emergence of vancomycin-resistant strains of *S. aureus* (VRSA) with vancomycin MIC value greater than 16 µg/ml [36],[41],[45].

## 3. Prevalence of MRSA and *S. aureus* with reduced sensitivity to vancomycin

MRSA has spread worldwide, and its prevalence has increased in both health-care and community environments. The proportion of MRSA varied among countries such as for instance: 0.4% in Sweden [24]; 25% in western part to 50% in southern India [10]; 33%–43% in Nigeria [46]; 37–56% in Greece, Portugal and Romania in 2014 [47]. High prevalence of MRSA with rates greater than 50% has also been reported in hospitals worldwide including in Asia, Malta, North and South America [29],[48]. Variation in the prevalence rates of MRSA was due to different epidemiological factors such as geographical and health system capability in running infection control program [49].

Akanbi and Mbe [50] reported a prevalence range of 0% to 6% VRSA in southern parts of Nigeria among clinical isolates and also 57.7% in Zaria, northern Nigeria. Goud, *et al*., [51] reported a vancomycin resistance in 1.4% of *S. aureus* isolates in southern India. Other countries such as: Australia, Korea, Hong Kong, Scotland, Israel, Thailand, South Africa, etc have also reported *S. aureus* with vancomycin sensitivity reduction with prevalence ranges from 0–74% [20],[36],[52].

## 4. Therapeutic measures

Currently, there are seven common antibiotics used against MRSA, which are: vancomycin, daptomycin, linezolid, Sulfamethoxazole and trimethoprim (TMP-SMZ), quinupristin-dalfopristin, clindamycin and tigecycline. These antibiotics are gradually losing their efficiency as MRSA strains are developing resistance against them [8],[20],[53]. Presently, the therapeutic alternatives available for treatment of infections caused by MRSA and *S. aureus* with reduced vancomycin susceptibility are limited. Therefore, there is a global urgency for the development of novel drugs that will be effective in the treatment of *S. aureus* exhibiting multidrug resistance so as to combat the scourge caused by the microorganism in the globe [52].

### 4.1. Prospects of medicinal plants as therapeutic option for MRSA

Natural products including medicinal plants have contributed immensely to human health, well-being and development of novel drugs. They are useful natural blueprints for the development of new drugs (especially in western countries) or/and phytomedicines purified to be used for the treatment of disease (commonly in developing countries and Europe) [54]. Medicinal plants can be valuable therapeutic resources. In numerous developing countries, including Nigeria, 80% of patients use home-made phytomedicines to treat infectious diseases. Despite the availability of modern medicine in some communities, the use of medicinal plants has remained high due to their efficacy, popularity and low cost. They also represent sources of potentially important new pharmaceutical substances since all the plants parts are utilized in traditional treatment and can therefore, act as lead compounds (Table 1).

The applications of phytomedicines for human well being and as blueprints for developing novel useful drugs have drastically increased worldwide in recent years [77].

The emergence of multidrug-resistant infectious agents associated with over- and inappropriate use of antibiotics has necessitated the World Health Organization (WHO) to acknowledge and pronounce the urgent need to develop novel antimicrobials and/or new approaches to tackle the menace caused by them in the globe; these have subsequently led to the resuscitation of the interest in medicinal plants [78]. The most common bacteria that have been used in susceptibility tests with numerous medicinal plants include: *Staphylococcus aureus*, methicillin-resistant *Staphylococcus aureus* (MRSA), vancomycin-resistant *Enterococcus* (VRE), *Pseudomonas aeruginosa Helicobacter pylori*, etc [54]. Presently, numerous studies have reported the antibacterial activity of many plant extracts against MRSA. In this study, only fifty-one (51) plants with anti-MRSA activities from thirty-five (35) families were mentioned (Table 1). The minimum inhibitory concentrations (MIC) values of the plants on the tested MRSA strains were between 1.25 µg/ml to 6.30 mg/ml. Twenty-nine of the plants had MIC values < 1.0 mg/ml while the remaining twenty-two MIC values were > 1.0 mg/ml but < 8.0 mg/ml. Extracts exhibiting activities with MIC values below 8 mg/ml are widely accepted to possess some antimicrobial activity while those with values below 1 mg/ml are considered noteworthy [77],[79]. However, most of the plants in this review were not tested on *S. aureus* strains with reduced vancomycin susceptibility.

The solvents used for the medicinal plants extraction in this review were ethanol and methanol (Table 1). This is probably because alcoholic extracts have higher antimicrobial activity than aqueous extracts. It has been reported that ethanolic extracts have higher antimicrobial activity than aqueous extracts because of the presence of higher amounts of polyphenols. They are more efficient in cell walls and seeds degradation causing polyphenols to be released from cells. Also, the enzyme polyphenol oxidase, degrades polyphenols in water extracts but is inactive in methanol and ethanol. Moreover, water is a better medium for the growth of microorganisms than ethanol [80].

Although, methanol is more polar than ethanol but it is not frequently used for plant extraction due to its cytotoxic nature that may give incorrect results [81].

Extracts of medicinal plants are rich in phytochemicals. Phytochemicals or secondary metabolites are natural protective agents biosynthesized by plants against external stress and pathogenic attack. They are crucial for plant defences and survival. They have been divided into several categories: phenolics, alkaloids, steroids, terpenes, saponins, etc. They exhibit other bioactivities such as antimutagenic, anticarcinogenic, antioxidant, antimicrobial, and anti-inflammatory properties and are therefore responsible for the medicinal potential of plants (Table 2). Hence, from this review, anti-MRSA plants have antibacterial effect on MRSA strains and other medicinal/therapeutic uses as depicted in Table 2.

**Table 1.** Medicinal plants with activities on methicillin-resistant *S. aureus* (MRSA).

Botanical name	Family	Local/Common name	Place of collection	Plant part used	Extracting Solvent	MIC /MBC (mg/ml) MRSA	MIC /MBC (mg/ml) VRSA	References
*Acacia catechu* (L. f.) Willd	Fabaceae	Cutch tree, black catechu	Thailand	Wood	Ethanol	1.6–3.2/25		55,56
*Garcinia mangostana L.*	Clusiaceae	Mangosteen	Thailand	Fruit shell	Ethanol	0.05–0.4/0.1–0.4		55,57
*Impatiens balsamina*	Balsaminaceae	Garden balsam	Thailand	Leaf	Ethanol	6.3/25		55,58
*Peltophorum ptercarpum* (DC.)	Fabaceae	Yellow flame tree	Thailand	Bark	Ethanol	0.1–0.8/6.3		55,59
*Psidium guajava* L.	Myrtaceae	Guava	Thailand	Leaf	Ethanol	0.2–1.6/6.3		55,60
*Punica granatum* L.	Punicaceae	Pomegranate	Thailand	Fruit shell	Ethanol	0.2–0.4/1.6–3.2		55,61
*Uncaria gambir* (Hunter) Roxb.	Rubiaceae	Gambier, White cutch	Thailand	Leaf, stem	Ethanol	0.4–0.8/3.2		55,62
*Walsura robusta*	Meliaceae	Bonlichu	Thailand	Wood	Ethanol	1.6–3.2/25		55,63
*Swietenia mahagoni*	Meliaceae	Mahagoni	Malaysia	Seed	Ethanol	0.2–0.78/0.78–1.56		64
*Tinospora crispa*	Menispermaceae	Patawali	Malaysia	Stem	Ethanol	0.4–0.78/0.78–1.56		64
*Butea monosperma* Lam.	Fabaceae	Flame-of-the-forest	India	Leaf	Ethanol	5.91/13.30	1.16/2.62	65
*Callistemon rigidus* R.Br.	Myrtaceae	Stiff bottlebrush	India	Leaf	Methanol	0.00125–0.08		66
*Acacia albida* Del.	Fabaceae	Gawo	Nigeria	Stem bark	Methanol	3.0/4.0		67
*Anchomanes difformis* Engl.	Araceae	Chakara	Nigeria	Roots	Methanol	4.0/5.0		67
*Boscia senegalensis* Del.	Capparidaceae	Anza	Nigeria	Roots	Methanol	5.0/6.0		67
*Moringa oleifera* Lam.	Moringacceae	Zogale	Nigeria	Leaf	Ethanol	4.0/5.0		67
*Mormodica basalmina* Linn.	Cucurbitaceae	Garahuni	Nigeria	Whole plant	Methanol	4.0/5.0		67
*Nymphaea lotus* Linn.	Nymphaeaceae	White lotus	Nigeria	Leaf	Ethanol	5.0–10.0/10.0–30.0	5.0–10.0/10.0–30.0	68
*Pavetta crassipes* K. Schum.	Rubiaceae	Gadau	Nigeria	Leaf	Methanol	4.0/5.0		67
*Phyllanthus amarus* Schum. Thonn.	Euphorbiaceae	Geron tsuntsaye	Nigeria	Whole plant	Methanol	4.0/5.0		67
*Vernonia blumeoides* Hook. f.	Asteraceae	Bagashi	Nigeria	Aerial part	Ethanol	4.0/5.0		67
*Curcuma xanthorrhiza*	Zingiberaceae	Java ginger	Indonesia	Rhizome	Ethanol	0.5/ND		69
*Kaempferia pandurata* Roxb.	Zingiberaceae	Temu kunci, fingerroot	Indonesia	Rhizome	Ethanol	0.3/ND		69
*Senna alata*	Fabaceae	Candle bush	Indonesia	Leaf	Ethanol	0.5/ND		69
*Mallotus yunnanensis* Pax et. Hoffm.	Euphorbiaceae	-	China	Tender Branches & leaves (TBL)	Ethanol	0.008–0.032/0.064–0.26		70
*Skimmia arborescens* Anders.	Rutaceae	Japanese skimmia	China	TBL	Ethanol	0.016–0.064/0.13–0.26		70
*Cyclobalanopsis austroglauca* Y.T. Chang	Fagaceae	Oak	China	TBL	Ethanol	0.016–0.064/0.13–0.51		70
*Manglietia hongheensis* Y.m Shui et. W.H. Chen.	Magnoliaceae	Magnolia	China	TBL	Ethanol	0.008–0.13/0.032–0.51		70
Brandisia hancei Hook.f.	Scrophulariaceae	-	China	Whole plant	Ethanol	0.032–0.064/0.13–0.26		70
*Evodia daneillii* (Benn) Hemsl.	Rutaceae	Bebe tree	China	TBL	Ethanol	0.032–0.064/0.064–0.26		70
*Schima sinensis* (Hemsl. et. Wils) Airy-shaw.	Theaceae	Schima	China	TBL	Ethanol	0.016–0.064/0.064–0.26		70
*Garcinia morella* Desr.	Clusiaceae	Gamboge	China	Whole plant	Ethanol	0.016–0.064/0.064–0.26		70
*Meliosma squamulata* Hance.	Lauraceae	-	China	TBL	Ethanol	0.032–0.064/0.13–0.26		70
*Curculigo orchioides* Gaertn.	Hypoxidaceae	Golden eye-grass	China	Whole plant	Ethanol	0.26–0.51/0.51–>2.05		70
*Euonymus fortunei* (Turcz.); Hand. Mazz.	Celastraceae	Spindle, Winter creeper	China	Vane	Ethanol	0.51/1.02–>2.05		70
*Alnus nepalensis* D. Don.	Betulaceae	Nepalese alder	China	TBL	Ethanol	0.26–1.02/1.02–>2.05		70
*Illicium simonsii* Maxim.	Illiciaceae	-	China	TBL	Ethanol	0.51–1.02/1.02–>2.05		70
*Blumea balsamifer* (Linn.) D.C.	Asteraceae	Sambong	China	Whole plant	Ethanol	0.064–0.26/0.26–1.02		70
*Machilus salicina* Hance.	Lauraceae	Liu ye run nan	China	TBL	Ethanol	0.51–1.02/1.02–>2.05		70
*Schisandra viridis* A.c.Smith.	Schisandraceae	Magnolia vine	China	Vane	Ethanol	0.064–0.26/0.26–1.02		70
*Selaginella tamariscina* (Seauv.) Spring.	Selaginellaceae	Little club moss	China	Whole plant	Ethanol	0.51–1.02/1.02–>2.05		70
*Celastrus orbiculatus* Thunb.	Celastraceae	Chinese bittersweet	China	Vane	Ethanol	0.51–1.02/1.02–>2.05		70
*Polygonum molle*D. Don.	Polygonaceae	Knotweed	China	Whole plant	Ethanol	0.26–0.51/1.02–>2.05		70
*Carex prainii* C.B. Clarke	Cyperaceae	Sedges	China	Whole plant	Ethanol	1.02–2.05/2.05–>2.05		70
*Embelia burm*f.	Myrsinaceae	Baberung, Vidanga	China	Leaves	Ethanol	0.51–1.02/1.02–>2.05		70
*Melianthus major* L.	Melianthaceae	Giant honey flower	South Africa	Leaves	Ethanol	0.78/3.12		71
*Melianthus comosus*Vahl	Melianthaceae	Honey flower	South Africa	Leaves	Ethanol	0.39/1.56		71
*Dodonaea angustifolia* (L.f.) Benth	Sapindaceae	Sticky hopbush, sand olive	South Africa	Leaves	Ethanol	0.59/1.17		71
*Withania somnifera* L.	Solanaceae	Ashwagandha, Winter cherry	South Africa	Roots & leaves	Ethanol	1.56/>6.25		71,72,73
*Quercus infectoria* Olivier	Fagaceae	“Machika or Oak galls	South Africa	Nutgalls	Ethanol	0.4–3.2/3.2–6.3		74
*Thymus vulgaris* L.	Lamiaceae	Thyme	Peru	Leaves	Essential oil	0.057/ND		75,76

Key: ND- Not done; MIC- Minimum inhibitory concentration; MBC- Minimum bactericidal concentration; VRSA- Vancomycin-resistant *S. aureus*

**Table 2.** Anti-MRSA plants with their phytochemical contents and medicinal uses.

Medicinal Plant	Phytochemical content	Medicinal uses	References
*Acacia catechu*	tannins, flavonoids, amino acids , saponins, triterpenoids	Cold, cough, diarrhea, piles,fever. ulcers, boils,etc	82,83
*Garcinia mangostana*	Xanthones and phenolics (tannins)	skin infections, wounds, dysentery, urinary disorders, cystitis and gonorrhoea	84,85
*Impatiens balsamina*	flavanoids, triterpenoids, glycosides, fatty acids & alkaloids	diuretic, emetic, laxative, demulcent and tonic	86
*Peltophorum ptercarpum*	fatty acids, amino acids, terpenoids, phenolics, flavonoids, alkaloids, steroids etc.	stomatitis, insomnia, skin troubles, constipation, ringworm, insomnia, dysentery, muscular pains, sores, and skin disorders	87
*Psidium guajava*	Tannins, Steroids, Alkaloids, glycosides, vitamins, carbohydrates	diarrhea, sore throat, vomiting, stomach upset, vertigo etc.	88
*Punica granatum*	Tannins, Alkaloids, glycosides, vitamins, carbohydrates, flavanoids, saponins, triterpenoids	sore throats, coughs, urinary infections, digestive disorders, skin disorders, arthritis, expel worms	89
*Uncaria gambir*	tannins, catechin, gambiriins	wounds and ulcers, fevers, headaches, gastrointestinal illnesses, bacterial/fungal infections, diarrhoea, sore throat	90,91
*Walsura robusta*	Sesquiterpenoid 10-nitro-isodauc-3-en-15-al, 10-oxo-isodauc-3-en-15-al	Antibacterial, antimicrobial, astringent, diarrhea	92,93
*Swietenia mahagoni*	Alkaloids, terpenoids, anthraquinone, cardiac glycosides, saponins, phenols, flavonoids, etc	Hypertension, diabetes, malaria, amoebiasis, cough, chest pain, tuberculosis, antibacterial	64,94
*Tinospora crispa*	Triterpenes, flavones o-glycosides (apigenine), picroretoside, berberine, palmatine, picroretine & resin	Fever, jaundice, hyperglycemia, wounds, intestinal worms, skin infections, antibacterial activity	64
*Butea monosperma*	Tannins, Saponins, Alkaloids, Glycosides, Carbohydrates	hepatoprotective, antidiabetic, antihelmintic, antimicrobial, antitumour, antiulcer, inflammatory diseases, wound healing, etc	65
*Callistemon rigidus*	Tannins & phenolic compounds, Lipids & fats, Steroids, Alkaloids, Saponins, Terpenoids	Treatment of cough, bronchitis and respiratory tract infections	66,95
*Acacia albida*	Alkaloids, tannins, saponins, phenols, flavonoids	respiratory infections, skin infections, digestive disorders, malaria and other fevers, toothache in humans and eye infections in livestock.	96
*Anchomanes difformis*	Alkaloids, tannins, saponins	cough, respiratory diseases, dysentery	97,98
*Boscia senegalensis*	Alkaloids, anthraquinone, cardiac glycosides, saponins, phenols, tannins, etc	Anticancer and ulcer swellings	98
*Moringa oleifera*	anthraquinone, cardiac glycosides, saponins, phenols, tannins, flavonoids	Asthma, eye infections, migraine, headache, febrifuge, abortifacient	98
*Mormodica basalmina*	resins, alkaloids, flavonoids, glycosides, steroids, terpenes, cardiac glycoside, saponins	anti-HIV, anti-plasmodial,anti-diarrheal, anti-septic, anti-bacterial, anti-viral, anti-inflammatory, anti-microbial, etc	99
*Nymphaea lotus*	phenols, tannins, saponins, alkaloids and steroids	aphrodisiac, anodyne, astringent, cardiotonic, sedative, analgesic and as anti-inflammatory agent.	68,100
*Pavetta crassipes*	flavonoids, sugars, tannins, saponins, glycosides, alkaloids and polyphenols	respiratory infections and abdominal disorders,gonorrhoeae,cough remedy	101,102
*Phyllanthus amarus*	lignans, flavonoids, hydrolysable tannins (ellagitannins), polyphenols, triterpenes, sterols and alkaloids.	used in the problems of stomach, genitourinary system, liver, kidney and spleen. It is bitter, astringent, stomachic, diuretic, febrifuge and antiseptic	103
*Vernonia blumeoides*	glycosides, saponins, alkaloids, tannins, flavonoids, steroids/terpenes	treatment of various human ailments including parasitic (malaria) and infectious diseases	104
*Curcuma xanthorrhiza*	Alkaloids, terpenoids, cardiac glycosides, saponins, phenols, flavonoids, coumarin	Treatment of liver damage, hypertension, diabetes, and cancer.	105,106
*Kaempferia pandurata*	Flavonoids, such as pinostrobin, pinocembrin, alpinetin, cardamonin, etc	Treatment of cough, stomach distended, diuretic, anti-anthelminthic, uterus inflammation, vaginal infection	107,108
*Senna alata*	flavonoids, tannic acid, anthocyanin, alkaloids, quercetin and coumarins	Antimicrobial, antifungal, ringworm, asthma, aphthous ulcers	109
*Mallotus yunnanensis*	Polyphenols, tannins, flavonoids, coumarins, various terpenoids	hepatitis, sore, otitis media, stomach and duodenal ulcer, enlarged spleen and boils swelling, hematuria leucorrhea and traumatic bleeding	70
*Skimmia arborescens*	alkaloids, coumarins, triterpenoids, phenols	HBV (skimmianine), rheumatoid, paralysisa, beriberi, and containing toxic substances	70
*Cyclobalanopsis austroglauca*	None	astringing sores, carbuncles, dysentery, hemostasis and vaginal discharge	70
*Manglietia hongheensis*	Alkaloids	vomiting, diarrhea, dysentery, constipation and geriatric hacking cough	70
*Brandisia hancei*	hydroxytyrosol derivatives and glycosides	jaundice, boils, swelling, tuberculosis injury, hematemesis, osteomyelitis, periostitis, rheumatism and pain	70
*Evodia daneillii*	alkaloids, flavonoid glycosides, flavaprin, limonoids	diarrhea, abdominal pain and vomiting	70
*Schima sinensis*	benzoquinone, tannins, phenols, lignans, flavonoids, triterpenoids	furuncle and swelling	70
*Garcinia Morella*	phenols (gambogic acid), flavonoids (xanthones),triterpenoids	wound rot, carbuncle, tinea, ulcer and sore, anthelminthic and containing toxic substances	70
*Meliosma squamulata*	Triterpenoids	scabies, carbuncle boils swollen poison, hemorrhoids, enterobiasis, beriberi, rheumatoid, and snake bite	70
*Curculigo orchioides*	triterpenoids, lignans, flavonoids, alkaloids, stereoids	diarrhea, ulcer, pus and muscles atrophy	70
*Euonymus fortune*	alkaloids, triterpenoids, flavonoids	chronic diarrhea, dysentery, dispersing blood stasis and traumatic bleeding	70
*Alnus nepalensis*	tannins, triterpenoids, flavonoids, phenols	bleeding of the nose, enteritis and dysentery	70
*Illicium simonsii*	terpenoids, lignans, flavonoids, phenols	scabies, bladder hernias, mixed cropping of edible spices and containing toxic substances	70
*Blumea balsamifera*	flavonoids, simple terpenoids	anti-rheumatism, ringworm and sores, dysentery, detoxification and snake bite	70
*Machilus salicina*	alkaloids, lignans	carbuncle, furunculosis and sore pain	70
*Schisandra viridis*	lignans, triterpenoids, organic acids	urticaria, herpes zoster, rheumatism and analgesia	70
*Selaginella tamariscina*	flavonoids, phenol glycosides, trehalose	inflammation, pharyngolaryngitis and bacteriostasis	70
*Celastrus orbiculatus*	sesquiterpene, flavonoids	dysentery, multiple abscess, Herpes zoster, detoxification, inflammatory, cellulites and snake bite	70
*Polygonum molle*	tannins, flavonoids, alkaloids	carbuncle, swollen abscess, fistula and scrofula	70
*Carex prainii*	alkaloids, polyphenols, flavonoids	antipyresis, diuretic and chyluria	70
*Embelia burm*	quinones, triterpenoids, flavonoids	heat clearing and detoxicating, pharyngitis, dysentery, diarrhea, furuncle ulcer, skin itching, swelling and pain of hemorrhoids, etc	70
*Melianthus major*	quercetin 3-*O*-β-galactoside-6-gallate, kaempferol 3-O-α-arabinopyranoside	wound healing and sores	71,110
*Melianthus comosus*	Triterpenoids	wound healing, sores, skin inflammation, snakebite	110,111,112
*Dodonaea angustifolia*	diterpenoids, flavonoids	skin infections and irritations, inflammation, tuberculosis and pneumonia	110,113
*Withania somnifera*	withanolides, alkaloids, chlorogenic acid, glycosides, glucose, tannins, and flavonoids	anti-inflammatory, antimicrobial, antitumour, anti-convulsant, sedative	72,73
*Quercus infectoria*	tannin, saponin, gallic acid and ellagic acid	hemorrhages, chronic diarrhea, dysentery, Skin disease, sore throat	114,115,116
*Thymus vulgaris*	alkaloids, carbohydrates and glycosides, flavonoid, resins, saponins, tannins, sterols and triterpenes	headache, fevers, ulcers, arthritis, microbial infections even cancers	117

The therapeutic properties of these medicinal plants obtained from their phytochemicals could be employed for drug development [118]. The antibacterial (anti-MRSA) activity of these plants is attributed to their phytochemical contents. For instance, flavonoids complex with bacterial cell wall, extracellular and soluble protein while tannins inactivate microbial adhesions, enzymes and cell envelop proteins [55],[67]–[69],[119].

Although, these anti-MRSA plants are likely promising candidates for drug development for MRSA infections, it has been reported that most plants contain potentially toxic, mutagenic, and/or carcinogenic substances. Therefore, it is highly recommended that medicinal plants undergo a critical sequential antimicrobial, pharmacological, and toxicology screening to ascertain their safety and selection as good candidates for novel drug development [77],[79],[120].

## 5. Conclusion

*S. aureus* is a common microorganism that is widely spread in the human population with many being asymptomatic carriers. It can also cause life-threatening infections and its strains have evolved into MRSA and strains with reduced vancomycin susceptibility (VISA, hVISA and VRSA). These strains cause infections and diseases that are either difficult to treat or resistant to the empiric antibiotics usually prescribed for treatment. The globe is running short of drugs/antibiotics available for therapy as a result of infections associated with this organism.

Many research studies have reported that some medicinal plants in different countries have anti-MRSA activities due to their phytochemical contents. These plants can be employed as alternative candidates for drug development to halt or/and control the infections of multi-drug resistant *S. aureus*. However, there is a need for further studies to adequately determine the safety and clinical efficacy of anti-MRSA plants to man.
